# Identification of a RelA/SpoT Homolog and Its Possible Role in the Accumulation of Astaxanthin in *Haematococcus pluvialis*

**DOI:** 10.3389/fpls.2022.796997

**Published:** 2022-02-09

**Authors:** Hui Jin, Yong Min Lao, Jin Zhou, Zhong Hua Cai

**Affiliations:** ^1^Shenzhen Public Platform for Screening and Application of Marine Microbial Resources, Shenzhen International Graduate School, Institute for Ocean Engineering, Tsinghua University, Shenzhen, China; ^2^School of Pharmaceutical Sciences, Sun Yat-sen University, Guangzhou, China; ^3^Institute for Advanced Study, Shenzhen University, Shenzhen, China

**Keywords:** environmental stresses, stringent response, ppGpp (guanosine 3′, 5′ bisdiphosphate), astaxanthin, *Haematococcus pluvialis*

## Abstract

A RelA/SpoT homolog, HpRSH, was identified in *Haematococcus pluvialis*. HpRSH was found to catalyze Mg^2+^-dependent guanosine tetraphosphate (ppGpp) synthesis and Mn^2+^-dependent ppGpp hydrolysis, respectively. The transcription of HpRSH was significantly upregulated by environmental stresses, such as darkness, high light, nitrogen limitation, and salinity stress. The intracellular ppGpp level was also increased when exposed to these stresses. In addition, the classical initiator of stringent response, serine hydroxamate (SHX), was found to upregulate the transcription of HpRSH and increase the level of ppGpp. Moreover, stringent response induced by SHX or environmental stresses was proven to induce the accumulation of astaxanthin. These results indicated that stringent response regulatory system involved in the regulation of astaxanthin biosynthesis in *H. pluvialis.* Furthermore, stringent response was unable to induce astaxanthin accumulation under dark condition. This result implied that stringent response may regulate astaxanthin biosynthesis in a light-dependent manner.

## Introduction

*Haematococcus pluvialis* is a unicellular biflagellate green microalga that inhabits in freshwater (Lorenz and Cysewski, 2000). Recently, this alga attracts considerable attention because it accumulates the highest content of astaxanthin (up to 4% by dry weight), which is a high-value carotenoid with excellent antioxidant activity (Han et al., 2013). Previous studies have proven that astaxanthin accumulation is occurring when algal cells are subjected to unfavorable environments, such as nutrient starvation, high light, and salt stress (Sarada et al., 2002). The accumulation of astaxanthin is generally believed to be a survival strategy of *H. pluvialis* under photo-oxidative stress or other adverse environmental conditions (Hagen et al., 1994; Hu et al., 2008). Astaxanthin confers *H. pluvialis* a remarkable ability to survive in extremely unfavorable environments (Hu et al., 2008; Li et al., 2008). Accompanied by the accumulation of astaxanthin, this alga also exhibits a dimorphic life cycle, mobile green vegetative cells in favorable environment and non-mobile red cyst cells in unfavorable environment (Droop, 1954; Zhang et al., 2017). During this process, *H. pluvialis* stops dividing, enters into a resting stage, and maintains a lower metabolic rate (Boussiba and Vonshak, 1991). Although many studies focus on the relationship between astaxanthin accumulation and environment stresses (Boussiba et al., 1999; Huir, 2000; Su et al., 2014), less is known about the mechanism of how environmental stresses lead to the transformation of morphology and physiology and induce the accumulation of astaxanthin in *H. pluvialis*.

To survive in changing environments, organisms have evolved many stressful response systems. Stringent response is one of the most important bacterial adaptive responses to deal with nutrient limitation, which was first reported in *Escherichia coli* (Magnusson et al., 2005; Boutte and Crosson, 2013). In addition to nutrient limitation, it also mediates bacterial adaptations to other environmental stresses, such as oxidative damage and acid shock (Wells and Gaynor, 2006; Khakimova et al., 2013; Brown et al., 2014; Irving et al., 2021). Although described for the first time in *E*. *coli*, stringent response broadly exists in plants, algae, *Drosophila*, and human (Kasai, 2002; Takahashi et al., 2004; Sun et al., 2010). When cells are faced with adverse environmental stresses, stringent response is initiated by a small signal molecular, guanosine tetraphosphate (ppGpp) and/or guanosine pentaphosphate (pppGpp), collectively denoted as (p)ppGpp (Potrykus and Cashel, 2008; Hauryliuk et al., 2015). Once stringent response is activated, bacteria will reallocate their resources from growth support to survival prolonged by reprogramming relative genes transcription (Kanjee et al., 2012; Hauryliuk et al., 2015). A growing number of studies indicated that (p)ppGpp contributes to the regulation of many aspects of bacterial biology, such as growth adaption in *E. coli* (Traxler et al., 2011), antibiotic production in *Bacillus subtilis* (Ochi and Ohsawa, 1984), morphological differentiation in *Mycobacterium smegmatis* (Ojha et al., 2000), sporulation in *Streptomyces* (Ochi, 1987), social behavior in *pseudomonas aeruginosa* (Van Delden et al., 2001), and fruit body development in *Myxococcus xanthus* (Harris et al., 1998). Likewise, ppGpp was also operative in plant and algae, with the identification of ppGpp synthase homologs in *Arabidopsis thaliana* (Der Biezen et al., 2000) and *Chlamydomonas reinhardtii* (Kasai, 2002), the detection of ppGpp in pea, wheat, rice, and spinach (Takahashi et al., 2004), and the investigation of its roles in regulation of cell physiology in *Synechococcus elongatus* (Puszynska and O’Shea, 2017), red alga (Imamura et al., 2018), and *Phaeodactylum tricornutum* (Avilan et al., 2021).

Stringent response plays critical roles in cellular adaptation to environmental stresses (Potrykus and Cashel, 2008). The question of whether bacterial stringent response mediates adaption to stresses and induces accumulation of astaxanthin in *H. pluvialis* was raised. To validate this possibility, we attempted to clone and characterize the homolog of RelA/SpoT enzyme in *H. pluvialis*. And then, the interrelationship between stringent response and astaxanthin accumulation was investigated in detail through mimicking stringent response using serine hydroxamate (SHX), which is a classical initiator of stringent response (Nguyen et al., 2011). Finally, we showed that the RelA/SpoT homolog in *H. pluvialis*, HpRSH, is a double function enzyme, catalyzing Mg^2+^-dependent ppGpp synthesis and Mn^2+^-dependent ppGpp hydrolysis. Stringent response could be initiated by SHX and some stresses, such as nitrogen starvation, salinity stress, high light, and darkness. Moreover, stringent response activated by stresses or SHX caused a remarkable accumulation of astaxanthin and induced the upregulation of astaxanthin biosynthesis-related genes. Meanwhile, it was found that stringent response failed in inducing astaxanthin accumulation under dark condition. These results indicated that stringent response system involved in the accumulation of astaxanthin in a light-dependent manner in *H. pluvialis*.

## Materials and Methods

### Algal Strain and Growth Conditions

*Haematococcus pluvialis* 797 strain was obtained from Institute of Hydrobiology, Chinese Academy of Sciences. The Blue-Green Medium (BG11, whose composition is shown in Supplementary Table 1) was used for the growth of *H. pluvialis.* Algae were cultured in conical flask containing 100 ml of growth medium under constant illumination. Cultures were incubated in an illumination incubator at 22°C and 20 μmol photons m^–2^ s^–1^ of light provided by HITACHI lamp (FH32EN, GY10q-9, HITACHI, Japan) under a 12-h light:12-h dark cycle and were shaken manually two times daily. Light intensities were measured by a photometer (LI-250A, LI-COR Inc., Lincoln, NE, United States).

### Stresses Treatment

*Haematococcus pluvialis* in the logarithmic growth phase was treated by different stresses. For dark stress, cells were cultured in a black chamber; for high light stress, algal cells were cultured under illumination of 200 μmol photons m^–2^ s^–1^; for salt stress, NaCl was added to fresh BG11 at a final concentration of 42 μM; for nitrogen (N^–^) starvation, algal cells were washed three times using nitrogen-free (without nitrate) BG11 medium and inoculated into nitrogen-free BG11. For SHX treatment, SHX was added to the culture at a final concentration of 1 g/L. For the transcriptional analysis of HpRSH, algal cells were collected and exposed to different stresses for 0, 1, 2, 3, 6, and 9 h or SHX for 15 min, 30 min, 1, 2, 6, and 9 h. For the transcriptional analysis of some key genes related to astaxanthin accumulation and the determination of astaxanthin accumulation, algal cells were collected after stress exposure for 4 and 72 h, respectively.

### Isolation of the Full-Length cDNA of *Hprsh* Gene

According to the sequence of bifunctional (p)ppGpp synthetase/guanosine-3′,5′-bis(diphosphate) 3′-pyrophosphohydrolase annotated in the transcriptome of *H. pluvialis* (GenBank: SRX765192), the ORF of *Hprsh* was amplified by PCR using eHpRelA F and eHpRelA R primers and RNA as template.

Based on the ORF sequence, two gene-specific primers (GSPs) were designed to amplify the 5′-end of *Hprsh* mRNA. 5′-RACE reactions were accomplished using the SMARTer^®^ RACE 5′/3′ Kit (Clontech, United States) and two pairs of GSPs designed. All manipulations were according to the user manual. The full-length cDNAs were finally isolated using GSPs complementary with the 5′- and 3′-ends of the *Hprsh* gene, respectively. The parameters of PCRs were set as follows: 95°C for 5 min; 30 cycles of 95°C for 30 s, 55°C for 30 s, and 72°C for 4 min 30 s, with a final extension at 72°C for 10 min. The amplified sequences were cloned into pMD19-T vector (TaKaRa, China) and sequenced.

The localization of HpRSH was predicted by ChloroP 1.1^1^ and WoLF PSORT^2^. Then, the truncate ORF was, respectively, cloned into pET-32a (+) vector between *Bam*HI and *Hin*dIII sites and into pUC19 between *Hin*dIII and *Eco*RI, generating pET-32a-*Hprsh* expression vector and pUC19-*Hprsh* for functional complementation in *E. coli*. Primes for *Hprsh* isolation are listed in Supplementary Table 2.

### Complementation Analysis of *Hprsh* in *E. coli*

The *E. coli* wild-type strain CF1648 and its derived strain CF1693 (relA^–^/spoT^–^) were prepared as transformation strains. The plasmid pUC19-*Hprsh* including the truncate ORF without the transit peptide of HpRSH was transformed into CF1693 and CF1648. As a control, empty vector plasmid was also transformed into CF1693 and CF1648, respectively. All strains were coated to LB and M9M (M9 mineral medium) plate, respectively, and grown at 37°C for 24 h.

### Protein Purification and Enzyme Assay

Recombinant protein was produced in *E. coli* BL-21(DE3) containing the plasmid pET-32a-*Hprsh*. Cells were pelleted by centrifugation at the speed of 12,000 × *g* for 2 min at 4°C. Enzyme extracts were then prepared by the Ni-NTA Spin Kit (QIAGEN, German) according to the user protocol and were eluted using 300 μl of PBS (50 mM NaH_2_PO_4_, 300 mM NaCl, pH 7.0) containing 500 mM imidazole. The PBS buffer was then exchanged to Tris–HCl buffer (0.1 M Tris–HCl, pH 8.0, 5 mM DTT, 1 mM EDTA) by using the Zeba Spin Desalting Columns and Plates, 7K MWCO (Thermo Fisher Scientific, United States). Protein extracts were quantitated at 25°C using the TaKaRa BCA Protein Assay Kit (TaKaRa, China). All steps were carried out at 4°C unless otherwise stated.

The synthetic or hydrolytic activities of HpRSH were assayed by a method described previously (Nanamiya et al., 2008; Ito et al., 2012). Briefly, for synthase activity assay, reaction mixture containing 50 μM GDP and 80 μM ATP was incubated with 5 mM Mg^2+^ in the presence of recombinant HpRSH protein at 37°C for 20 min. For hydrolase activity, reaction mixture containing 50 mM Tris–HCl (pH 8.0), 5 mM Mn^2+^, 50 μM ppGpp, and 0.2–1.0 mg of the purified recombinant protein was incubated at 37°C for 20 min. The mixture was analyzed by UPLC on a HILIC chromatographic column (BEH Amide, Waters, United States). ppGpp and GDP were detected by their UV absorbance at 252 nm as reaction products of synthetic and hydrolytic activity, respectively.

### Phylogenic Analysis

Sequence analysis was performed using BLAST Software^3^. Multiple alignments were conducted using Clustal X version 1.83. Molecular evolutionary analysis was conducted using the Neighbor-Joining method by the molecular evolution genetics analysis (MEGA) software, version 5.2. Bootstrap values were estimated (with 1,000 replicates) to assess the relative support for each branch, and they were labeled with a cutoff value of 50.

### Extraction, Purification, and Detection of Guanosine Tetraphosphate

The analysis of ppGpp in algal cells was according to our published method (Jin et al., 2018). Notably, 2.4 g of cells were collected by centrifugation (8,000 × *g*, 10 min), frozen immediately in liquid nitrogen, crushed, and resuspended in 10 ml of 1 M cool formic acid. The broken cells were vigorously mixed, incubated on ice for 30 min, and then centrifuged (10,000 × *g*, 10 min). The upper layer was transferred into a new tube. The all supernatants were purified by solid-phase extract (SPE). First, the SPE column (OASIS@ WAX 3-cc Vac Cartridges, Waters) was pretreatment using 1 ml of MeOH and then 1% HCOOH. The supernatants were loaded immediately to SPE column. Then, the column loaded with supernatants was washed using 1 ml of 1% HCOOH and 1 ml of MeOH, respectively. Finally, the crude extracts were eluted by 1 ml of H_2_O/NH_4_OH (70:30) solution. The effluent was dried under vacuum. The resultant residue was dissolved in 100 μl H_2_O/ACN (25/75, v/v), and 5 μl was detected using UPLC-MS/MS.

All MS analysis were performed on a Waters TQ-XS triple quadrupole mass analyzed connected to a Waters Aquity H-class UPLC (Waters, United States), and data were collected and analyzed using the MassLynx version 4.1 software (Waters, United States). Chromatographic separation was performed on a Waters BEH Amide column (2.1 × 50 mm, 1.7 μm) at the flow rate of 0.4 ml min^–1^. The mobile phase was comprised by solvent A (0.1% NH_4_OH) and solvent B (acetonitrile). The gradient was set according to the following profile: 0.0 min, 20% A + 80% B; 4.0 min, 25% A + 75% B; 13.0 min, 25% A + 75% B; 14.0 min 50% A + 50% B; 17.0 min, 50% A + 50% B; 17.5 min, 80% A + 20% B. The injection volume was 5 μl. MS was operated in negative electrospray ionization (ESI) and in multiple reactions monitoring (MRM) mode. The ion resource settings were as follows: capillary voltage = 2.5 kV; cone voltage = 30 V; desolvation gas flow = 1,200 L h^–1^; cone gas flow = 150 L h^–1^; nebulizer gas = 7.0 bar; desolvation temperature = 400°C; source temperature = 150°C. The ion transition at *m*/*z* 602/159 was used for ppGpp detection. The cone voltage (V) and collision energy (eV) are 44 V and 44 eV, respectively.

### RT-PCR Analysis

For real-time quantitative reverse transcription PCR (qPCR) analysis, 30 ml of cells was collected by centrifugation at 12,000 × *g* for 2 min. Total RNA was isolated and purified using the TRIzol according to the manufacturer’s instructions. Real-time qPCR was performed with a 7500 Real-Time PCR System (Applied Biosystems, United States) using PrimeScript^®^ RT reagent Kit with gDNA Eraser (which supplies RNase-free DNase I to remove any co-isolated genomic DNA) and SYBR Green PCR Kit (product code: DRR041A and DRR047A, respectively; TaKaRa, China). Primers for quantitative analysis are listed in Supplementary Table 2.

The reaction mix contained 4 μl of cDNA, 0.5 μl of forward and reverse primer mix (20 μM each), 1 μl of 50 × ROX Reference Dye II and 25 μl 2 × TaKaRa SYBR Green PCR mix in a final volume of 50 μl. All reactions were set up in triplicate, and every sample was replicated in parallel three times to ensure statistical relevance. The following standard thermal conditions were used for all PCR reactions: 30 s at 95°C, 40 cycles of 30 s at 95°C, and 34 s at 60°C. Primer specificity was confirmed by RT-PCR amplification before Real-Time Quantitative PCR reaction, which produced single amplicon of the expected size for each primer set; these amplicons were sequenced to finally validate their specific amplification. The specificity of qPCR reaction was monitored by the presence of dissociation curves with single peak and sequencing of its products with unique bands of the expected size. Amplicon dissociation curves were obtained after cycle 40 with default settings suggested by the instrument. Data were analyzed using the SDS software (Applied Biosystems, United States).

### Astaxanthin Extraction, Saponification, and Detection

Algal cells were collected by centrifugation at 12,000 × *g* for 15 min. The process of extraction, saponification, and detection was according to our published method (Jin et al., 2017). In brief, 100 ml of algae cells (OD_600_ at 0.45) were collected by centrifugation at 8,000 × *g* for 10 min at 25°C, lyophilized and weighed, sequentially. The freeze-dried cells were resuspended in 10 ml of 1 M cool chloroform and sonicated at 4°C for 5 min. The broken cells were vigorously mixed, incubated on ice for 30 min, and then centrifuged at 12,000 × *g* at 4°C for 15 min. The upper layer was collected. Second extraction was carried out, and the supernatant was then merged, evaporated to dryness, and dissolved with 5 ml acetonitrile. The extract was stored at -80°C until use. Then, 100 μl extract was diluted with 2 ml acetonitrile and hydrolyzed by adding 10 μl 1 M NaOH. The saponification process lasted for 6 h at 4°C in dark. The saponified extract was then washed several times with distilled water until the pH was neutral and analyzed by UPLC directly.

Carotenoids were separated at a flow rate of 0.4 ml min^–1^ on Waters BEH C18 column (2.1 × 50 mm, 1.7 μm), using mobile phases composed of methanol (A) and acetonitrile (B). The gradient elution was 10% A and 90% B at 0 min, followed by linear gradient to 0% A and 100% B to 4 min, maintained at 0% A and 100% B to 12 min, returned to initial condition by 12.1 min, re-equilibrated at initial condition by 15 min. The injection volume was 5 μl. Needle was washed using acetonitrile/methanol (9:1; v/v) mixture for 10 s after each injection. Column temperature was maintained at 35°C using a column oven. The detection of analysts was carried out by UV absorbance at 450 nm. The filter constant was set at 0.2.

### Statistical Analysis

The analysis of variance was performed on SPSS software. Two-tailed Student’s *t*-test (refer to Figures 3–5) was performed with assumptions of a Gaussian distribution and equal variance. The number of degrees of freedom for all tests was 4, for *n* = 3 in each set.

## Results

### Functional Identification of HpRSH

**FIGURE 1:**
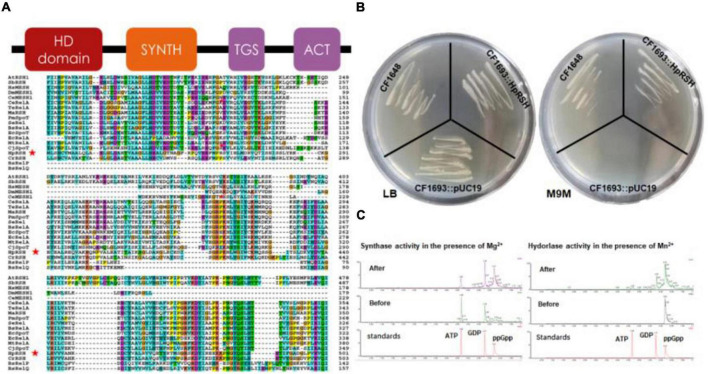
Characterization of relA/spoT homologous gene *Hprsh* in *H. pluvialis*. **(A)** Secondary conserved domains and sequence alignment of MaRSH. Red stars indicate the sequence of HpRSH. **(B)** Functional complementation of HpRSH. **(C)** *In vitro* activities of HpRSH. Left: In the presence of Mg^2+^, the enzyme utilized ATP and GDP as substrates (before the reaction, middle panel) to synthesize ppGpp (after the reaction, upper panel). Right: In the presence of Mn^2+^, HpRSH displayed hydrolase activity to hydrolyze ppGpp (before the reaction, middle panel) into GDP (after the reaction, upper panel).

TblastN against the *H. pluvialis* transcriptome by the *C. reinhardtii* RSH (CrRSH) retrieved a HpRSH candidate. Based on this sequence, RT-PCR was performed and a predicted 2,409 bp ORF was isolated. Then, 3′- and 5′-RACE reactions were conducted to isolate the full-length cDNA (Supplementary Figure 1). Results showed that the full-length *Hprsh* mRNA has a 166 bp 5′-UTR and 1,140 bp 3′-UTR, with an ORF of 2,409 bp encoding a polypeptide with 796 aa (Figure 1A). It has typical secondary structures of RelA/SpoT superfamily, e.g., HD domain (hydrolase), RelA_SpoT domain (synthase), TGS domain, and ACT domain (Figure 1A). These results implied that HpRSH may be a RelA/SpoT-like enzyme. Using ChloroP and WoLF PSORT, HpRSH is predicted to contain a 59 aa long transit peptide and, therefore, is localized in chloroplast.

The function of HpRSH was determined by functional complementation in *E. coli* strains CF1693 (relA^–^spoT^–^) and CF1648 (wild type). According to previous research, the high level of ppGpp is lethal for *E. coli* due to the lack of (p)ppGpp hydrolase activity (Xiao et al., 1991). Furthermore, it should be noted that (p)ppGpp^0^ cell are unable to grow in nutritionally poor M9M medium because suitable concentration of ppGpp is needed for transcription of several operons involved in amino acid biosyntheses (Xiao et al., 1991; Ingraham et al., 1996). In this study, as shown in Figure 1B, on M9M plate, CF1693 with the pUC19 (empty vector) cannot grow, but transcribing the pUC19-HpRSH (carrying the full length of HpRSH) into CF1693 can restore the growth on M9M plate. These results indicate that HpRSH could complement relA^–^spoT^–^ phenotypes and function as an RSH, i.e., as a (p)ppGpp synthase and hydrolase bifunctional enzyme.

To further confirm the activity of HpRSH, we next examined *in vitro* synthetic and hydrolytic activities using recombinant protein (Supplementary Figure 2) at 35°C under different reaction condition (as shown in the section “Protein Purification and Enzyme Assay”) and analyzed reaction products through UPLC. As expected, ppGpp was found when incubating recombinant protein with ATP and GDP at the presence of Mg^2+^ as cofactor, and GDP was detected in the mixture of hydrolytic assay which contained Mn^2+^ (Figure 1C), demonstrating HpRSH catalyzes Mg^2+^-dependent ppGpp synthesis and Mn^2+^-dependent ppGpp hydrolysis.

### Molecular Evolutionary Relationships of HpRSH

**FIGURE 2:**
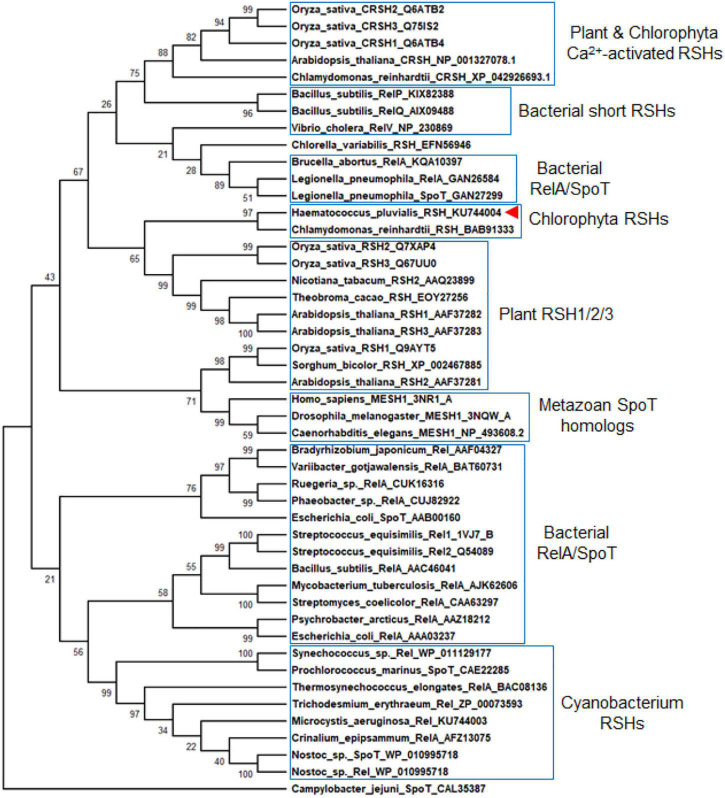
Molecular evolutionary relationships of HpRSH by the Neighbor-Joining method. The nodes of gene duplication during evolution are dotted. The bootstrap consensus tree inferred from 1,000 replicates is taken to represent the evolutionary history of the taxa analyzed. Branches corresponding to partitions reproduced in less than 50% bootstrap replicates are collapsed. The percentage of replicate trees in which the associated taxa clustered together in the bootstrap test (1,000 replicates) are shown above the branches. Red arrow indicates the HpRSH isolated in this study.

Previous studies found that *C. reinhardtii* has a single RSH (CrRSH, GenBank: EFN56946) (Kasai, 2002); it does not cluster within any other plant RSH (Boniecka et al., 2017); CRSH appears to be present only in land plants (Tozawa et al., 2007). However, we used this CrRSH, AtRSH1-3 (GenBank: AAF37282, AAF37281, AAF37283, respectively), AtCRSH (GenBank: NP_001327078.1), and HpRSH (GenBank: KU744004) characterized here to tblastn search the newest version (v5.6) of C. *reinhardtii* genome^4^ and found another RSH member, CrCRSH (GenBank: XP_042926693.1), with high query cover and confident score (Supplementary Material Phytozome BLAST file). Then, we used these RSHs, including CrRSH, CrCRSH, AtRSH1-3, and AtCRSH, to search the transcriptome of *H. pluvialis*, only the current HpRSH was retrieved. Based on the current HpRSH, a phylogenetic tree was constructed using RSH homologs. Results showed that HpRSH clustered with CrRSH, which was independent of other clusters of RSHs (Figure 2).

**FIGURE 3:**
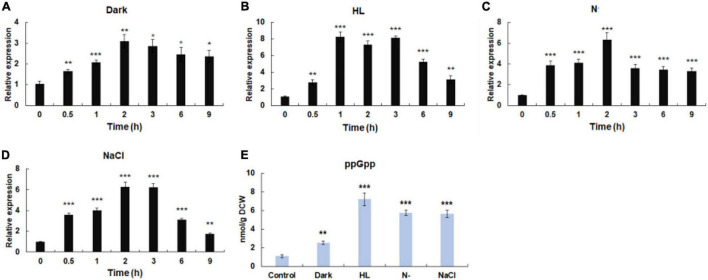
Influence of environmental stresses on the initiation of stringent response. **(A)** The transcriptional expressions of *Hprsh* under different stresses. **(B)** The levels of ppGpp induced by different stresses. Dark, keep in dark place; HL, high light; N^–^, nitrogen limitation (without nitrate sodium); NaCl, 42 μm NaCl; DCW, dry cell weight. Error bars indicate the standard deviation (SD) of the mean (*n* = 3) for *Hprsh* transcription analysis, or *n* = 3 for ppGpp determination. *p < 0.05, **p < 0.01, or ***p < 0.001 vs. 0 h **(A–D)** or control **(E)**.

**FIGURE 4:**
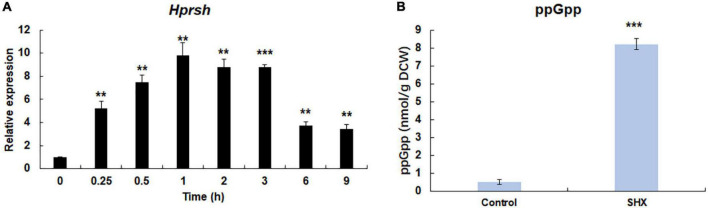
SHX-triggered stringent response. **(A)** The transcription of *Hprsh* induced by SHX. **(B)** The levels of ppGpp induced by different stresses at the highest transcriptional level of *Hprsh* (1 h for SHX). DCW: dry cell weight. Error bars indicate the standard deviation (SD) of the mean (*n* = 3) for the transcription of *Hprsh* and ppGpp determination. **p < 0.01, or ***p < 0.001 vs. 0 h **(A)** or control **(B)**.

**FIGURE 5:**
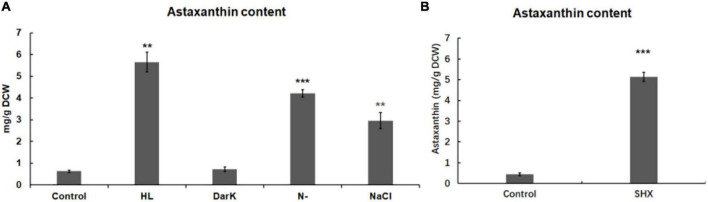
Effect of environmental stresses and SHX on the accumulation of astaxanthin in *H. pluvialis*. The accumulation of astaxanthin induced by environmental stresses **(A)** and SHX (1 mg/ml) for 72 h **(B)**. DCW: dry cell weight. Error bars indicate the standard deviation (SD) of the mean (*n* = 3) for *Hprsh* transcription analysis or *n* = 3 for astaxanthin determination. **p < 0.01, or ***p < 0.001 vs. control.

### Stringent Response Could Be Initiated by Environmental Stresses

Previous studies have shown that stressful conditions such as light, nutrient limitation, and salinity stress could induce stringent response (Takahashi et al., 2004). Moreover, it was proven that environmental factors induce the expression of RSH in a time-dependent manner (Jin et al., 2020). Therefore, time-course expression profiles of HpRSH under different stresses were investigated in this study. As shown in Figure 3A, when algal cells were exposed to darkness, the transcription of HpRSH was upregulated gradually and rose to maximum level (about 3.1-fold, *p* < 0.01) after 2 h. High light induced a significant increase of the transcription of HpRSH (2.8-fold, *p* < 0.01) after 30 min and then resulted in the highest level (about 8.3-fold, *p* < 0.001) at 1 h (Figure 3B). Similarly, the transcription of HpRSH was raised to more than 6-fold at 2 h by nitrogen limitation and salinity stress, respectively (Figures 3C,D). In addition, since alarmone ppGpp is the arbiter of stringent response, its corresponding concentration at the highest transcriptional level of HpRSH (1 h for high light and 2 h for all other groups) was determined to further confirm the initiation of stringent response biochemically. As expected, the endogenous ppGpp level under environmental stresses was consistent with the transcription of HpRSH. As exhibited in Figure 3E, when subjected to darkness, high light, nitrogen starvation, and salt stress, the concentration of ppGpp reached to a level of 2.54 ± 0.19 (*p* < 0.001), 7.22 ± 0.68 (*p* < 0.001), 5.77 ± 0.30 (*p* < 0.001), and 5.58 ± 0.39 (*p* < 0.001) nmol/g DCW (dry cell weight), respectively, compared with that of control (1.11 ± 0.13 nmol/g DCW). These results show that environmental stresses could trigger stringent response in *H. pluvialis.*

### Serine Hydroxamate Triggers Stringent Response in *H. pluvialis*

It is well-known that under amino acids starvation, uncharged tRNAs signal RelA to initiate stringent response in *E. coli* (Potrykus and Cashel, 2008). SHX, an inhibitor of serine tRNA synthetase, has been proven to elicit stringent response by simulating amino acid starvation in *E. coli*, *P. aeruginosa*, and some other bacteria (Van Delden et al., 2001; Potrykus and Cashel, 2008). To test whether SHX has the same effect on *H. pluvialis*, SHX was added to the algal culture media at a final concentration of 1.0 mg ml^–1^ and time-course transcriptional expression profile of HpRSH was determined in this study. As shown in Figure 4A, the transcription of HpRSH was upregulated after just 15 min, which reached the highest level at 1 h (about 9.8-fold, *p* < 0.01). Correspondingly, at the highest transcription level of HpRSH, the intracellular concentration of ppGpp was found to increase from 0.51 ± 0.12 at 0 h to 8.20 ± 0.31 nmol/g DCW (*p* < 0.001) at 1 h (Figure 4B). These results suggested that SHX was able to trigger stringent response in *H. pluvialis* and could be used as a positive control in this study.

### Higher Guanosine Tetraphosphate Level Enhanced Astaxanthin Accumulation

In this study, some environmental stresses were found to elicit stringent response by upregulating the transcription of HpRSH and enhancing ppGpp synthesis in *H. pluvialis*. These stresses had been proven to induce astaxanthin accumulation in previous studies (Kobayashi et al., 1993; Boussiba et al., 1999; Boussiba, 2000; Sarada et al., 2002). This observation led to the hypothesis that stringent response involves in the regulation of astaxanthin biosynthesis. To evaluate the effect of stressful conditions on the accumulation of astaxanthin, the levels of astaxanthin under different stresses were determined by UPLC. As shown in Figure 5A, environment stresses could differentially regulate astaxanthin biosynthesis. Except for darkness, stresses led to astaxanthin accumulation when compared with the control. Among them, high light presented the most significant effect on astaxanthin accumulation. It enhanced the yield of astaxanthin to 5.65 ± 0.45, compared with 0.63 ± 0.05 mg/g DCW in the control. Nitrogen limitation could also raise the level of astaxanthin obviously to 4.22 ± 0.17 mg/g DCW. The addition of NaCl resulted in an increase of astaxanthin to 2.96 ± 0.37 mg/g DCW. This result indicated that with the exception of darkness, stressful conditions initiated stringent response and concomitantly led to astaxanthin accumulation, which implied a connection between the initiation of stringent response and the accumulation of astaxanthin.

To confirm this possibility, SHX was used to elicit stringent response, then the content of astaxanthin was determined. As depicted in Figure 5B, the content of astaxanthin was strikingly accumulated from 0.45 ± 0.07 to 5.13 ± 0.21 mg/g DCW. Furthermore, there was a marked change in algal morphology. As depicted in Supplementary Figure 3, when exposed to 1 mg ml^–1^ SHX, algae transformed from green motile cells to red non-motile cells after 10 days. Oppositely, the control cells were with almost no changes. These results further demonstrated that stringent response may be involved in the regulation of astaxanthin biosynthesis.

### Stringent Response Induced the Upregulation of the Key Genes for Astaxanthin Biosynthesis

**FIGURE 6:**
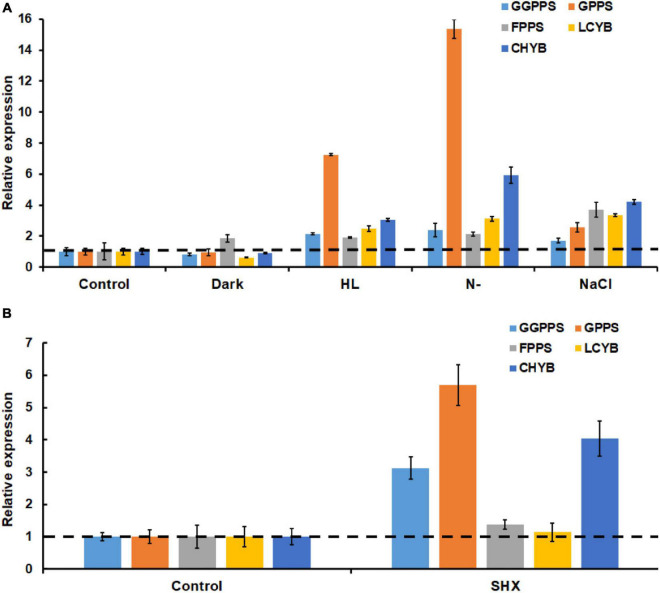
Transcriptional expressions of key astaxanthin biosynthesis genes under SHX **(A)** and different environmental stresses **(B)**. CHYB, carotenoid β-hydroxylase; LCYB, lycopene ε-cyclase; GPPS, geranyl diphosphate synthase; FPPS, farnesyl diphosphate synthase; and GGPPS, geranylgeranyl diphosphate synthase.

It was well-documented that environmental stresses caused the accumulation of astaxanthin through upregulating astaxanthin biosynthesis genes at the transcriptional level (Steinbrenner and Linden, 2003; Vidhyavathi et al., 2008). In this study, the transcription of general carotenogenic genes (e.g., *GPPS*, *FPPS*, and *GGPPS*) and specific astaxanthin synthesis genes (e.g., *LCYB* and *CHYB*) during stress exposure was investigated. As expected, both general carotenogenic genes and specific astaxanthin biosynthetic genes were found to be upregulated upon exposure to high light, nitrogen limitation, and salinity stress. Notably, 7.3- and 15.4-fold increases in the transcript of GGPPS were induced by nitrogen limitation and high light, respectively (Figure 6A). Meanwhile, both nitrogen limitation and high light resulted in more than 2-fold increase in the transcripts of GPPS, FPPS, LCYB, and CHYB (Figure 6A). In addition, salinity stress increased significantly the transcription of all the five genes. Darkness only upregulated the transcription of GGPP. Similarly, when stringent response was triggered by SHX, the expression levels of GGPPS, GPPS, FPPS, LCYB, and CHYB were found to be 4. 04-, 5. 69-, 1. 38-, 1. 14-, and 3.13-fold higher than the control group, respectively (Figure 6B). These results suggested environmental stresses (except darkness) upregulated several key genes in astaxanthin biosynthesis by initiating stringent response.

## Discussion

### Stringent Response May Be Involved in the Regulation of Astaxanthin Biosynthesis

Stringent response is highly conserved and regulates many important physiological functions in plant and algae (Kasai, 2002; Takahashi et al., 2004; Avilan et al., 2021). In this study, homologous protein of RelA/SpoT in *H. pluvialis*, HpRSH, was identified by function complement and enzyme assay *in vitro* (Figure 1). Both the transcription of *Hprsh* and the levels of ppGpp were stimulated by environmental stresses (Figure 3). In addition, a classic initiator of stringent response, SHX, was proven to elicit stringent response in *H. pluvialis* (Figure 4). These results suggested that stringent response system exists and possibly participates in the physiological regulation respond to stresses in *H. pluvialis*. Moreover, these environmental stresses were shown to stimulate *H. pluvialis* to produce astaxanthin (Figure 4A) and, meanwhile, SHX-activated stringent response to induce massive accumulation of astaxanthin under normal environment (Figure 4B). These results suggested that stringent response may be involved in the regulation of astaxanthin biosynthesis. The positive regulation of many key genes in astaxanthin biosynthesis by environmental stresses and SHX further validated the possibility (Figure 6). Astaxanthin accumulation is regarded as a responsive pathway against stresses to protect algal cells from damages under unfavorable conditions. Stringent response has been proven to be indispensable for organisms to adapt to diverse unfavorable environments (Boussiba, 2000). Moreover, stringent response is highly conserved and lies at the top network that governs global gene expression in response to environmental stresses (Hauryliuk et al., 2015). Therefore, on the basis of previous reports and our results, this study postulated that astaxanthin accumulation may be a consequence of stringent response induced by unfavorable environments. Algal cells sense stresses through stringent response, transmit this signal by ppGpp, and copy with stressful condition by redirection of transcription so that genes important for survival are favored, i.e., astaxanthin synthesis genes in this study.

### Astaxanthin Accumulation Mediated by Stringent Response May Be Light-Dependent

Our results showed that stringent response may be involved in the regulation of astaxanthin biosynthesis in *H. pluvialis*. It is worthy noted that darkness triggered a clear stringent response, but it failed to induce astaxanthin accumulation (Figures 3A, 5A). The role of light in astaxanthin biosynthesis has been studied in previous studies. Goodwin and Jamikorn claimed that light is necessary for astaxanthin biosynthesis (Goodwin and Jamikorn, 1954). Droop showed that astaxanthin could be synthesized in dark only when culture medium contains sodium acetate (Droop, 1955). According to these points, it is reasonable that darkness was unable to induce astaxanthin accumulation in culture medium without sodium acetate, although it did initiate stringent response. These results may imply that stringent response regulates astaxanthin biosynthesis in a light-dependent manner. Actually, SHX-activated stringent response was also unable to induce astaxanthin accumulation under dark condition in culture medium without sodium acetate (Supplementary Figure 4), which further strengthens the light-dependent regulation hypothesis by stringent response.

## Conclusion

In this study, HpRSH, a RelA/SpoT homolog, was identified by functional complement and *in vitro* enzyme activity in *H*. *pluvialis*. HpRSH was found to possess double functions and catalyzes Mg^2+^-dependent ppGpp synthesis and Mn^2+^-dependent ppGpp hydrolysis. Both the transcription of HpRSH and the level of ppGpp were raised by SHX and environmental stresses, such as darkness, nitrogen starvation, high light, and salinity stress. Moreover, stringent response induced by SHX or environmental stresses led to a significant accumulation of astaxanthin. Furthermore, key genes in astaxanthin biosynthesis were upregulated by SHX and these stresses. All these results showed that stringent response may be involved in the regulation of astaxanthin biosynthesis in *H*. *pluvialis*. In addition, darkness triggered stringent response but was unable to induce astaxanthin accumulation. Meanwhile, SHX-activated stringent response was unable to cause astaxanthin accumulation under dark condition. These results implied that the regulation of astaxanthin biosynthesis by stringent response may be light-dependent.

## Data Availability Statement

The datasets presented in this study can be found in online repositories. The names of the repository/repositories and accession number(s) can be found in the article/Supplementary Material.

## Author Contributions

HJ and YL contributed to conceptualization, methodology, investigation, writing – original draft and review and editing, and funding acquisition. JZ contributed to data analysis, validation, investigation, and writing – review and editing. ZC contributed to conceptualization, investigation, writing – review and editing, supervision, project administration, and funding acquisition. All authors contributed to the article and approved the submitted version.

## Conflict of Interest

The authors declare that the research was conducted in the absence of any commercial or financial relationships that could be construed as a potential conflict of interest.

## Publisher’s Note

All claims expressed in this article are solely those of the authors and do not necessarily represent those of their affiliated organizations, or those of the publisher, the editors and the reviewers. Any product that may be evaluated in this article, or claim that may be made by its manufacturer, is not guaranteed or endorsed by the publisher.

## Footnotes

1
https://services.healthtech.dtu.dk/service.php?ChloroP-1.1

2
https://wolfpsort.hgc.jp/

3
http://blast.ncbi.nlm.nih.gov/

4
https://phytozome-next.jgi.doe.gov/
