# Supplementary material for: Identification of a RelA/SpoT Homolog and Its Possible Role in the Accumulation of Astaxanthin in Haematococcus pluvialis
Source: Front Plant Sci. 2022 Feb 9;13:796997. doi: 10.3389/fpls.2022.796997 (PMC8863741; doi:10.3389/fpls.2022.796997)
Supplement: Supplementary file 1 [file Data_Sheet_1.PDF]

TBLASTN 2.2.29+

Reference: Stephen F. Altschul, Thomas L. Madden, Alejandro A. Schaffer, Jinghui Zhang, Zheng Zhang, Webb Miller, and David J. Lipman (1997), "Gapped BLAST and PSI-BLAST: a new generation of protein database search programs", Nucleic Acids Res. 25:3389-3402.

Database: Creinhardtii_281.genome
54 sequences; 111,100,715 total letters

Query= AtRSH2_AAF37281

Length=883

Sequences producing significant alignments:	Score (Bits)	E Value	
jgi|chromosome_3 Creinhardtii|281|v5.5	85.9	3e-16	CrRSH
jgi|chromosome_14 Creinhardtii|281|v5.5	57.8	1e-07	

>jgi|chromosome_3 Creinhardtii|281|v5.5
Length=9219486

Score = 85.9 bits (211), Expect = 3e-16, Method: Compositional matrix adjust.
Identities = 60/223 (27%), Positives = 108/223 (48%), Gaps = 43/223 (19%)
Frame = -1

```
Query  137
PSISYLPRKELEFVQKGLKLAFEAHHGQKRRSGEPFIIHPVAV-----------------  179
               P  +  P    E +      LA  AH  Q+R++GE  + H VAV
Sbjct  2504844
PGQAEFPIFNNELLVHAYGLAARAHQNQQRKNGESLLSHCVAVSCCGVGWCGRVRVAMA
S  2504665

Query  180     --------------------ARILGELELDWESIVAGLLHDTVEDTNFITFEKIEEEFGA
219
                                   A+  +   L LD E++ A LLH+ V+     ++   ++EE
Sbjct  2504664
RGLPRHSNLTHGFIGYTRQVAKTVAGLGLDAETVAAALLHECVD---MVSRSQLEEFMPP
2504494
```

Query 220
TVRHIVEGETKVSKLGKLKCKTESETIQDVKADDLRQMFLAMTDEVRVIIVKLADRLHNM
279
            +V ++E   +S++ +L   +   +D+ Q L   ++V+ +IVKL+DRL +
Sbjct 2504493
SVVSVLEHVNTISEMSRLY---RQHSATGSFSDETFQRLLVGFEDVKAVIVKLSDRLAEL
2504323

Query 280   RTLCHMPPHKQSSIAGETLQVFAPLAKLLGMYSIKSELENLSF 322
            RT+ +P +Q+++A ETL+V++ +A LG++ +K+ELE+L+F
Sbjct 2504322 RTISVLPADRQAALARETLEVYSVVANRLGVWCLKAELEDLAF 2504194

Score = 45.8 bits (107), Expect = 5e-04, Method: Compositional matrix adjust.
Identities = 17/28 (61%), Positives = 21/28 (75%), Gaps = 0/28 (0%)
Frame = -2

Query 436   IWKPIPRTVKDYIATPKPNGYQSLHTTV 463
            +W+PIP   KDYI PK NGYQS+H T+
Sbjct 8940227 LWRPIPSRSKDYITAPKTNGYQSIHLTL 8940144

Score = 41.2 bits (95), Expect = 0.012, Method: Compositional matrix adjust.
Identities = 19/39 (49%), Positives = 24/39 (62%), Gaps = 1/39 (3%)
Frame = -1

Query 426   CYHVLGLVHEIWKPIPRTVKDYIA-TPKPNGYQSLHTTV 463
            CY L V +++ +P   KD+I   KPNGYQSLH TV
Sbjct 2503776 CYTALRAVQSVYRVMPSRSKDFIKDIKKPNGYQSLHETV 2503660

>jgi|chromosome_14 Creinhardtii|281|v5.5
Length=4157777

Score = 57.8 bits (138), Expect = 1e-07, Method: Compositional matrix adjust.
Identities = 23/38 (61%), Positives = 28/38 (74%), Gaps = 0/38 (0%)
Frame = +3

Query 424   QICYHVLGLVHEIWKPIPRTVKDYIATPKPNGYQSLHT 461
            + CY ++ VH IWKPI R   DYIA PKP+GYQ+LHT
Sbjct 1980288 EACYRLVSAVHSIWKPIKREFDDYIANPKPSGYQALHT 1980401

```
Lambda      K        H
   0.319    0.133    0.398

Gapped
Lambda      K        H
   0.267   0.0410    0.140

Effective search space used: 28325807235


Query= AtRSH1_AAF37282

Length=710
                                                                      Score     E
Sequences producing significant alignments:                          (Bits)  Value

jgi|chromosome_3  Creinhardtii|281|v5.5                                152   3e-37   CrRSH
jgi|chromosome_14  Creinhardtii|281|v5.5                               47.0    2e-04


>jgi|chromosome_3 Creinhardtii|281|v5.5
Length=9219486

 Score =   152 bits (384),  Expect = 3e-37, Method: Compositional matrix adjust.
 Identities = 123/466 (26%), Positives = 207/466 (44%), Gaps = 108/466 (23%)
 Frame = -1

Query  141
LDGSFRLGASRL-FNGFVRKALGSCVDYELGSDSGLVDELTFPMEVDTIKPYARDLLRRA  199
              L  S  LG  R   +G +R  LG C      + S L      P  ++   +P+ R
Sbjct  2504985
LQDSGWLGLRRARLSGMLRGNLGCC------NSSSLDVGCAIPAAIN--RPFLRG---PG
2504839

Query  200
QLRHKIFNDESVIKAFYEAEKAHRGQMRASRDPYLQHCV---------------------  238
              Q    IFN+E ++ A+   A +AH+ Q R + +  L HCV
Sbjct  2504838
QAEFPIFNNELLVHAYGLAARAHQNQQRKNGESLLSHCVAVSCCGVGWCGRVRVAMASR
G  2504659

Query  239
----------------ETAMLLANIGANSTVVVAGLLHDTVDDSFMSYDYILRNFGAGVA  282
                            + A  +A +G ++  V A LLH+ VD   +S   +       V
Sbjct  2504658
```

LPRHSNLTHGFIGYTRQVAKTVAGLGLDAETVAAALLHECVD--MVSRSQLEEFMPPSVV
2504485

Query 283
DLVEGVSKLSQLSKLARENNTACKTVEADRLHPMFLAMADARAVLIKLADRLHNMKTLYA
342
++E V+ +S++S+L R++ +A + + + + D +AV++KL+DRL ++T+
Sbjct 2504484
SVLEHVNTISEMSRLYRQH-SATGSFSDETFQRLLVGFEDVKAVIVKLSDRLAELRTISV
2504308

Query 343
LSPVKQQRFAKETLEIFAPLANCLGISTWKVQLENLCFKHLYPN---------------- 386
L +Q A+ETLE+++ +AN LG+ K +LE+L F L+P
Sbjct 2504307
LPADRQAALARETLEVYSVVANRLGVWCLKAELEDLAFSVLHPQVRGACAGSFLCMIRDA
2504128

Query 387 ---------------------------------QHNEMSTMLEDSFDEAMITSAIEKLD
412
++N + + D + +AI +
Sbjct 2504127
RV*VLRLAAPLLLPLYGPRIWR*SVCLLIHVNLQEYNWLKEAVRTRQDPVALETAINSIK
2503948

Query 413
QALKKAGISYHVLCGRHKSLYSIYSKMLK--KKLTVDEI---HDIHGLRLIVDNEGDCYK 467
AL+ G++Y + GR K+LY I+ K+ K K LT++ + +D+ LR++V ++ +CY
Sbjct 2503947
GALQAQGVAYEDISGRPKNLYGIFMKLQKDGKPLTIESLNSLYDLMALRVVVAHKHECYT
2503768

Query 468 ALGVVHSLWSEVPGKLKDYITH-PKFNGYQSLHTVVMDNGTVPLEV 512
AL V S++ +P + KD+I K NGYQSLH V G VP+EV
Sbjct 2503767 ALRAVQSVYRVMPSRSKDFIKDIKKPNGYQSLHETVYGEGDVPVEV
2503630

Score = 47.0 bits (110), Expect = 2e-04, Method: Compositional matrix adjust.
Identities = 21/30 (70%), Positives = 25/30 (83%), Gaps = 1/30 (3%)
Frame = -3

Query 507 TVP-LEVQIRTQEMHLQAEFGFAAHWRYKE 535
TVP +VQIRT +MH AE+GFAAHW+YKE
Sbjct 2503465 TVPRAQVQIRTHKMHYIAEYGFAAHWKYKE 2503376

```
 Score = 44.7 bits (104),  Expect = 8e-04, Method: Compositional matrix adjust.
 Identities = 17/29 (59%), Positives = 21/29 (72%), Gaps = 0/29 (0%)
 Frame = -2

Query  471      VVHSLWSEVPGKLKDYITHPKFNGYQSLH  499
                V  +LW  +P + KDYIT PK NGYQS+H
Sbjct  8940239  VASTLWRPIPSRSKDYITAPKTNGYQSIH  8940153


>jgi|chromosome_14 Creinhardtii|281|v5.5
Length=4157777

 Score = 47.0 bits (110),  Expect = 2e-04, Method: Compositional matrix adjust.
 Identities = 20/36 (56%), Positives = 25/36 (69%), Gaps = 0/36 (0%)
 Frame = +2

Query  500      TVVMDNGTVPLEVQIRTQEMHLQAEFGFAAHWRYKE  535
                + V   G +P+EVQI+T  MH  AE+G AAHW YKE
Sbjct  1980722  SAVRGPGGIPMEVQIKTSSMHELAEYGAAAHWVYKE  1980829


 Score = 45.8 bits (107),  Expect = 4e-04, Method: Compositional matrix adjust.
 Identities = 17/36 (47%), Positives = 25/36 (69%), Gaps = 0/36 (0%)
 Frame = +3

Query  465      CYKALGVVHSLWSEVPGKLKDYITHPKFNGYQSLHT  500
                CY+ +  VHS+W  +  + DYI +PK +GYQ+LHT
Sbjct  1980294  CYRLVSAVHSIWKPIKREFDDYIANPKPSGYQALHT  1980401


 Score = 38.1 bits (87),  Expect = 0.089, Method: Compositional matrix adjust.
 Identities = 15/41 (37%), Positives = 25/41 (61%), Gaps = 0/41 (0%)
 Frame = +3

Query  204      KIFNDESVIKAFYEAEKAHRGQMRASRDPYLQHCVETAMLL  244
                + F   +V+ A   A  AH  Q R + +PY+ HC+ETA+++
Sbjct  1973361  RFFRIPAVLAAVEFAAVAHAAQRRKTGEPYVTHCIETALIV  1973483



Lambda      K        H
   0.319    0.133    0.398
```

Gapped
Lambda K H
0.267 0.0410 0.140

Effective search space used: 28325807235

Query= AtRSH3_AAF37283

Length=712

Sequences producing significant alignments:	Score (Bits)	E Value
jgi|chromosome_3 Creinhardtii|281|v5.5	155	2e-38
jgi|chromosome_14 Creinhardtii|281|v5.5	50.1	2e-05

CrRSH

>jgi|chromosome_3 Creinhardtii|281|v5.5
Length=9219486

Score = 155 bits (393), Expect = 2e-38, Method: Compositional matrix adjust.
Identities = 115/455 (25%), Positives = 207/455 (45%), Gaps = 111/455 (24%)
Frame = -1

```
Query  154
FNGFVRKAIGSCVDYDTDSVLVDEQLPFTMDDGFEGERRQPYARDLLRRAQLKHKIFEDE
213
           +G +R  +G C   ++ S+ V   +P  ++  F    R P        Q +  IF +E
Sbjct  2504946
LSGMLRGNLGCC---NSSSLDVGCAIPAAINRPF---LRGP--------GQAEFPIFNNE
2504809

Query  214     SVIKAFYEAEKAHRGQMRATGDPYLQHCV------------------------------
242
           ++ A+  A +AH+ Q R  G+  L HCV
Sbjct  2504808
LLVHAYGLAARAHQNQQRKNGESLLSHCVAVSCCGVGWCGRVRVAMASRGLPRHSNLT
HG  2504629

Query  243
------ETAMLLADIGANSTVVVAGILHDTLDDSFMSYDYILRTFGSGVADLVEGVSQLS  296
              + A  +A +G ++  V A +LH+ +D   +S  +      V  ++E V+ +S
Sbjct  2504628
FIGYTRQVAKTVAGLGLDAETVAAALLHECVD--MVSRSQLEEFMPPSVVSVLEHVNTIS
2504455
```

Query 297
KLAR--ENNTACKTVEADRLHTMFLAMADARAVLIKLADRLHNMMTLYALPPVKRQRFAK
354
+++R   ++A +   +   + +   D +AV++KL+DRL + T+ LP ++   A+
Sbjct 2504454
EMSRLYRQHSATGSFSDETFQRLLVGFEDVKAVIVKLSDRLAELRTISVLPADRQAALAR
2504275

Query 355   ETLEIFAPLANRLGISSWKVKLENLCFKHLHPD---------------------------
387
ETLE+++ +ANRLG+   K +LE+L F LHP
Sbjct 2504274
ETLEVYSVVANRLGVWCLKAELEDLAFSVLHPQVRGACAGSFLCMIRDARV*VLRLAAPL
2504095

Query 388
-----------------------QHHEMSDMLEDSFDEAMITSAIEKLEQALKKEGISYH 424
+++ + + +   D   + +AI ++ AL+ +G++Y
Sbjct 2504094
LLPLYGPRIWR*SVCLLIHVNLQEYNWLKEAVRTRQDPVALETAINSIKGALQAQGVAYE
2503915

Query 425
VVSGRHKSLYSIYCKMLK--KKLT---MDEIHDIHGLRLIVDNEKDCYKALGVVHKLWSE 479
+SGR K+LY I+ K+ K K LT   ++ ++D+ LR++V ++ +CY AL V ++
Sbjct 2503914
DISGRPKNLYGIFMKLQKDGKPLTIESLNSLYDLMALRVVVAHKHECYTALRAVQSVYRV
2503735

Query 480   VPGKLKDYISH-PKFNGYQSLHTVVMGDGTIPLEV 513
+P + KD+I   K NGYQSLH V G+G +P+EV
Sbjct 2503734 MPSRSKDFIKDIKKPNGYQSLHETVYGEGDVPVEV 2503630

Score = 46.2 bits (108), Expect = 3e-04, Method: Compositional matrix adjust.
Identities = 20/30 (67%), Positives = 25/30 (83%), Gaps = 1/30 (3%)
Frame = -3

Query 508   TIP-LEVQIRTKEMHLQAEFGFAAHWRYKE 536
T+P +VQIRT +MH AE+GFAAHW+YKE
Sbjct 2503465 TVPRAQVQIRTHKMHYIAEYGFAAHWKYKE 2503376

Score = 43.1 bits (100), Expect = 0.003, Method: Compositional matrix adjust.

Identities = 18/36 (50%), Positives = 24/36 (67%), Gaps = 1/36 (3%)
Frame = -2

Query 472   VVHKLWSEVPGKLKDYISHPKFNGYQSLH-TVVMGD 506
              V   LW +P + KDYI+ PK NGYQS+H T+ +G
Sbjct 8940239 VASTLWRPIPSRSKDYITAPKTNGYQSIHLTLQLGQ 8940132

>jgi|chromosome_14 Creinhardtii|281|v5.5
Length=4157777

Score = 50.1 bits (118), Expect = 2e-05, Method: Compositional matrix adjust.
Identities = 22/36 (61%), Positives = 26/36 (72%), Gaps = 0/36 (0%)
Frame = +2

Query 501   TVVMGDGTIPLEVQIRTKEMHLQAEFGFAAHWRYKE 536
              + V G G IP+EVQI+T MH AE+G AAHW YKE
Sbjct 1980722 SAVRGPGGIPMEVQIKTSSMHELAEYGAAAHWVYKE 1980829

Score = 44.7 bits (104), Expect = 9e-04, Method: Compositional matrix adjust.
Identities = 16/36 (44%), Positives = 25/36 (69%), Gaps = 0/36 (0%)
Frame = +3

Query 466   CYKALGVVHKLWSEVPGKLKDYISHPKFNGYQSLHT 501
              CY+ + VH +W + + DYI++PK +GYQ+LHT
Sbjct 1980294 CYRLVSAVHSIWKPIKREFDDYIANPKPSGYQALHT 1980401

Score = 42.4 bits (98), Expect = 0.004, Method: Compositional matrix adjust.
Identities = 17/41 (41%), Positives = 26/41 (63%), Gaps = 0/41 (0%)
Frame = +3

Query 208   KIFEDESVIKAFYEAEKAHRGQMRATGDPYLQHCVETAMLL 248
              + F   +V+ A   A AH Q R TG+PY+ HC+ETA+++
Sbjct 1973361 RFFRIPAVLAAVEFAAVAHAAQRRKTGEPYVTHCIETALIV 1973483

Lambda   K   H
0.319   0.133   0.398

Gapped
Lambda   K   H
0.267   0.0410   0.140

Effective search space used: 28325807235

Query= AtCRSH_NP_001327078.1

Length=445

Score E

Sequences producing significant alignments: (Bits) Value

jgi|chromosome_3 Creinhardtii|281|v5.5 87.0 2e-17 CrRSH & CrCRSH
jgi|chromosome_14 Creinhardtii|281|v5.5 44.3 5e-04

>jgi|chromosome_3 Creinhardtii|281|v5.5
Length=9219486

Score = 87.0 bits (214), Expect = 2e-17, Method: Compositional matrix adjust.
Identities = 90/336 (27%), Positives = 147/336 (44%), Gaps = 94/336 (28%)
Frame = -1

Query 126
LQMDAEVISASILSEVVDANA---------ISIYEVRDHIGTGTAHLLHEIFRVKNIPFK 176
L +DAE ++A++L E VD + S+ V +H+ T + E+ R+
Sbjct 2504586
LGLDAETVAAALLHECVDMVSRSQLEEFMPPSVVSVLEHVNT-----ISEMSRLYRQHSA
2504422

Query 177
VDVLDDETAASLRKFYLTYYDIRAVIMDLVSKLDEMRHLDHLPRYRQQILSLEVLKIYSP 236
DET ++ + + D++AVI+ L +L E+R + LP RQ L+ E L++YS
Sbjct 2504421
TGSFSDET---FQRLLVGFEDVKAVIVKLSDRLAELRTISVLPADRQAALARETLEVYSV
2504251

Query 237
LAHAVGANHLSLELEDISFRYLFPCSYIYLDSWLRGHENGS------------------- 277
+A+ +G L ELED++F L P +RG GS
Sbjct 2504250
VANRLGVWCLKAELEDLAFSVLHP--------QVRGACAGSFLCMIRDARV*VLRLAAPL
2504095

Query 278 -----KP---------LIDVYKEQLH------RSLKDDLVLAEMVNDVY-----------
306
P LI V ++ + R+ +D + L +N +

Sbjct 2504094
LLPLYGPRIWR*SVCLLIHVNLQEYNWLKEAVRTRQDPVALETAINSIKGALQAQGVAYE
2503915

Query 307
-IKGRYKSRYSMMKKLLRDGRKP---EEVN---DVLGLRVILMPNSVVNDVEVGEKACYR 359
I GR K+ Y + KL +DG KP E +N D++ LRV++ + CY
Sbjct 2503914
DISGRPKNLYGIFMKLQKDG-KPLTIESLNSLYDLMALRVVV----------AHKHECYT
2503768

Query 360 TSEIIRSLWKEIPHRTKDYIAR-PKENGYRSLHMAV 394
++S+++ +P R+KD+I K NGY+SLH V
Sbjct 2503767 ALRAVQSVYRVMPSRSKDFIKDIKKPNGYQSLHETV 2503660

Score = 57.0 bits (136), Expect = 6e-08, Method: Compositional matrix adjust.
Identities = 37/102 (36%), Positives = 61/102 (60%), Gaps = 5/102 (5%)
Frame = -3

Query 89 LFKALKLSIPILQSLPLASDGRsplskalslsiilaDL-----QMDAEVISASILSEVVD
143
+ ALKL++ LQ+ P DGRSP ++A+ L+ LADL +DA + A +++E D
Sbjct 8943274
IITALKLALSSLQASPPRRDGRSPATRAVQLASTLADLCAAGLPLDAFAMCAGVVAEAAD
8943095

Query 144 ANAISIYEVRDHIGTGTAHLLHEIFRVKNIPFKVDVLDDETA 185
A+ +R +G A L+H++ RV+ P ++++LDDE A
Sbjct 8943094 VGALRADVIRAQLGPSVAALVHDMLRVRQAPRRIELLDDEGA 8942969

Score = 51.2 bits (121), Expect = 4e-06, Method: Compositional matrix adjust.
Identities = 18/45 (40%), Positives = 34/45 (76%), Gaps = 0/45 (0%)
Frame = -1

Query 186 ASLRKFYLTYYDIRAVIMDLVSKLDEMRHLDHLPRYRQQILSLEV 230
+++R++ L ++D+RA +++V DE++H+ LP+Y QQ L+LEV
Sbjct 8942793 SAVREWCLAFHDVRACAVEVVCWWDELQHMGGLPQYEQQALALEV
8942659

Score = 47.4 bits (111), Expect = 6e-05, Method: Compositional matrix adjust.
Identities = 16/37 (43%), Positives = 26/37 (70%), Gaps = 0/37 (0%)
Frame = -2

```
Query  362      EIIRSLWKEIPHRTKDYIARPKENGYRSLHMAVDVSD  398
                ++  +LW+ IP R+KDYI  PK NGY+S+H+ + +
Sbjct  8940242  QVASTLWRPIPSRSKDYITAPKTNGYQSIHLTLQLGQ  8940132


 Score = 39.3 bits (90),  Expect = 0.018, Method: Compositional matrix adjust.
 Identities = 18/33 (55%), Positives = 26/33 (79%), Gaps = 1/33 (3%)
 Frame = -2

Query  402      IRPL-MEIQIRTMDMDGSANAGTASHSLYKGGL  433
                + PL +E+QIRT  MD +A +G A+H++YKGGL
Sbjct  8939792  VGPLCLELQIRTAAMDRAAESGDAAHAVYKGGL  8939694


>jgi|chromosome_14 Creinhardtii|281|v5.5
Length=4157777

 Score = 44.3 bits (103),  Expect = 5e-04, Method: Compositional matrix adjust.
 Identities = 18/39 (46%), Positives = 24/39 (62%), Gaps = 0/39 (0%)
 Frame = +3

Query  355      KACYRTSEIIRSLWKEIPHRTKDYIARPKENGYRSLHMA  393
                +ACYR    + S+WK I     DYIA PK +GY++LH
Sbjct  1980288  EACYRLVSAVHSIWKPIKREFDDYIANPKPSGYQALHTG  1980404



Lambda      K        H
   0.319    0.133    0.398

Gapped
Lambda      K        H
   0.267   0.0410    0.140

Effective search space used: 28325807235


Query= HpRSH_KU744004

Length=796
                                                                      Score     E
Sequences producing significant alignments:                          (Bits)  Value

jgi|chromosome_3  Creinhardtii|281|v5.5                                313   4e-90   CrRSH
```

jgi|chromosome_14 Creinhardtii|281|v5.5 51.6 6e-06

>jgi|chromosome_3 Creinhardtii|281|v5.5
Length=9219486

Score = 313 bits (803), Expect = 4e-90, Method: Compositional matrix adjust.
Identities = 177/405 (44%), Positives = 235/405 (58%), Gaps = 95/405 (23%)
Frame = -1

Query 188
QERHAVFHTDLVCTAYMLASKAHAGQHRKDGTSMLSHCV--------------------- 226
Q +F+ +L+ AY LA++AH Q RK+G S+LSHCV
Sbjct 2504838
QAEFPIFNNELLVHAYGLAARAHQNQQRKNGESLLSHCVAVSCCGVGWCGRVRVAMASR
G 2504659

Query 227
----------------MAALQLAELGLDAETVAAGLLHEALGTNAGFRPQVEEFMPRSVI 270
A +A LGLDAETVAA LLHE + + R Q+EEFMP SV+
Sbjct 2504658
LPRHSNLTHGFIGYTRQVAKTVAGLGLDAETVAAALLHECVDMVS--RSQLEEFMPPSVV
2504485

Query 271
HLMDRVQTISEISSLYRK--CKGQLGEEKMRRMLLAMEDVKAMLIKLACRVHDMKTVAAL
328
+++ V TISE+S LYR+ G +E +R+L+ EDVKA+++KL+ R+ +++T++ L
Sbjct 2504484
SVLEHVNTISEMSRLYRQHSATGSFSDETFQRLLVGFEDVKAVIVKLSDRLAELRTISVL
2504305

Query 329
PRDAQISLAQETLDIYSVVANRLGIWSLKAELEDLAFAVLHP------------------ 370
P D Q +LA+ETL++YSVVANRLG+W LKAELEDLAF+VLHP
Sbjct 2504304
PADRQAALARETLEVYSVVANRLGVWCLKAELEDLAFSVLHPQVRGACAGSFLCMIRDAR
2504125

Query 371 --------------------------------EECEALREQVAKRQNPVALEATITAIKS
398
+E L+E V RQ+PVALE I +IK
Sbjct 2504124
V*VLRLAAPLLLPLYGPRIWR*SVCLLIHVNLQEYNWLKEAVRTRQDPVALETAINSIKG
2503945

Query 399
GLDARGLRCEDISGRPKNLWGIWCKMQSSG----ITSLDKVYDVTALRVVVANKHDCYVA
454
L A+G+ EDISGRPKNL+GI+ K+Q G I SL+ +YD+ ALRVVVA+KH+CY A
Sbjct 2503944
ALQAQGVAYEDISGRPKNLYGIFMKLQKDGKPLTIESLNSLYDLMALRVVVAHKHECYTA
2503765

Query 455 LRVVQETYRTMSGRSKDYIREEKKPNGYQSLHETIFGADGLPVEV 499
LR VQ YR M RSKD+I++ KKPNGYQSLHET++G +PVEV
Sbjct 2503764 LRAVQSVYRVMPSRSKDFIKDIKKPNGYQSLHETVYGEGDVPVEV
2503630

Score = 140 bits (352), Expect = 4e-33, Method: Compositional matrix adjust.
Identities = 75/145 (52%), Positives = 96/145 (66%), Gaps = 2/145 (1%)
Frame = -2

Query 531
LDKEVQYKKWLMTYKLGVHDKKVRATGAPKQDCALTSLGMHLLDSATGSADAVGVDPFLR
590
L +E QYK+WL YKL VHDKKVR G+P D +L SLG+ +D + +DPFL+
Sbjct 2503220
LLQETQYKRWLTQYKLRVHDKKVRPQGSPPTDSSLKSLGVAYMDMPEEQQRGL-LDPFLQ
2503044

Query 591
HDRFKLGVPTKQRVSVVVATQDGIDAKELPANQSAQQLSDDLLGPRGLPGYTMTVNRRIP
650
H+RFKL VP K VSV++ T DGI+ K+ P +A QL D LG PGY +TVN R+P
Sbjct 2503043
HERFKLQVPAKTEVSVLLQTCDGIETKDFPLGTTANQLWRD-LGLGAQPGYALTVNSRLP
2502867

Query 651 APEYLLQTGDLVQVLPLAQALSQMP 675
+ E LQ+GDLVQVLPL+ LS+ P
Sbjct 2502866 SGEAALQSGDLVQVLPLSTILSRSP 2502792

Score = 74.7 bits (182), Expect = 6e-13, Method: Compositional matrix adjust.
Identities = 29/37 (78%), Positives = 35/37 (95%), Gaps = 0/37 (0%)
Frame = -3

Query 497 VEVQIRTHKMHFIAEYGFAAHWKYKEKLPNEDEWLDK 533

+VQIRTHKMH+IAEYGFAAHWKYKE++ +EDEWL+K
Sbjct 2503453 AQVQIRTHKMHYIAEYGFAAHWKYKEQMDSEDEWLEK 2503343

>jgi|chromosome_14 Creinhardtii|281|v5.5
Length=4157777

Score = 51.6 bits (122), Expect = 6e-06, Method: Compositional matrix adjust.
Identities = 22/37 (59%), Positives = 27/37 (73%), Gaps = 0/37 (0%)
Frame = +2

Query 489 IFGADGLPVEVQIRTHKMHFIAEYGFAAHWKYKEKLP 525
+ G G+P+EVQI+T MH +AEYG AAHW YKE P
Sbjct 1980728 VRGPGGIPMEVQIKTSSMHELAEYGAAAHWVYKEYTP 1980838

Lambda K H
0.319 0.133 0.398

Gapped
Lambda K H
0.267 0.0410 0.140

Effective search space used: 28325807235

Query= CrRSH_BAB91333

Length=735

Sequences producing significant alignments:	Score (Bits)	E Value	
jgi|chromosome_3 Creinhardtii|281|v5.5	610	0.0	CrRSH
jgi|chromosome_14 Creinhardtii|281|v5.5	50.4	2e-05	

>jgi|chromosome_3 Creinhardtii|281|v5.5
Length=9219486

Score = 610 bits (1573), Expect = 0.0, Method: Compositional matrix adjust.
Identities = 316/404 (78%), Positives = 316/404 (78%), Gaps = 87/404 (22%)
Frame = -1

Query 185

AQAEFPIFNNELLVHAYGLAARAHQNQQRKNGESLLSHCVAV------------------ 226
QAEFPIFNNELLVHAYGLAARAHQNQQRKNGESLLSHCVAV
Sbjct 2504841
GQAEFPIFNNELLVHAYGLAARAHQNQQRKNGESLLSHCVAVSCCGVGWCGRVRVAMAS
R 2504662

Query 227
-------------------AKTVAGLGLDAETVAAALLHECVDMVSRSQLEEFMPPSVVS 267
AKTVAGLGLDAETVAAALLHECVDMVSRSQLEEFMPPSVVS
Sbjct 2504661
GLPRHSNLTHGFIGYTRQVAKTVAGLGLDAETVAAALLHECVDMVSRSQLEEFMPPSVVS
2504482

Query 268
VLEHVNTISEMSRLYRQHSATGSFSDETFQRLLVGFEDVKAVIVKLSDRLAELRTISVLP 327

VLEHVNTISEMSRLYRQHSATGSFSDETFQRLLVGFEDVKAVIVKLSDRLAELRTISVLP
Sbjct 2504481
VLEHVNTISEMSRLYRQHSATGSFSDETFQRLLVGFEDVKAVIVKLSDRLAELRTISVLP
2504302

Query 328
ADRQAALARETLEVYSVVANRLGVWCLKAELEDLAFSVLHP------------------- 368
ADRQAALARETLEVYSVVANRLGVWCLKAELEDLAFSVLHP
Sbjct 2504301
ADRQAALARETLEVYSVVANRLGVWCLKAELEDLAFSVLHPQVRGACAGSFLCMIRDARV
2504122

Query 369 -------------------------------QEYNWLKEAVRTRQDPVALETAINSIKGA
397
QEYNWLKEAVRTRQDPVALETAINSIKGA
Sbjct 2504121
*VLRLAAPLLLPLYGPRIWR*SVCLLIHVNLQEYNWLKEAVRTRQDPVALETAINSIKGA
2503942

Query 398
LQAQGVAYEDISGRPKNLYGIFMKLQKDGKPLTIESLNSLYDLMALRVVVAHKHECYTAL
457

LQAQGVAYEDISGRPKNLYGIFMKLQKDGKPLTIESLNSLYDLMALRVVVAHKHECYTAL
Sbjct 2503941
LQAQGVAYEDISGRPKNLYGIFMKLQKDGKPLTIESLNSLYDLMALRVVVAHKHECYTAL
2503762

Query 458 RAVQSVYRVMPSRSKDFIKDIKKPNGYQSLHETVYGEGDVPVEV 501

RAVQSVYRVMPSRSKDFIKDIKKPNGYQSLHETVYGEGDVPVEV
Sbjct 2503761 RAVQSVYRVMPSRSKDFIKDIKKPNGYQSLHETVYGEGDVPVEV
2503630

Score = 373 bits (958), Expect = 3e-111, Method: Compositional matrix adjust.
Identities = 201/203 (99%), Positives = 202/203 (99%), Gaps = 0/203 (0%)
Frame = -2

Query 533
LEKETQYKRWLTQYKLRVHDKKVRPQGSPPTDSSLKSLGVAYMDMPEEQQRGLLDPFLQH
592
L
+ETQYKRWLTQYKLRVHDKKVRPQGSPPTDSSLKSLGVAYMDMPEEQQRGLLDPFLQH
Sbjct 2503220
LLQETQYKRWLTQYKLRVHDKKVRPQGSPPTDSSLKSLGVAYMDMPEEQQRGLLDPFLQH
2503041

Query 593
ERFKLQVPAKTEVSVLLQTCDGIETKDFPLGTTANQLWRDLGLGAQPGYALTVNSRLPSG
652

ERFKLQVPAKTEVSVLLQTCDGIETKDFPLGTTANQLWRDLGLGAQPGYALTVNSRLPSG
Sbjct 2503040
ERFKLQVPAKTEVSVLLQTCDGIETKDFPLGTTANQLWRDLGLGAQPGYALTVNSRLPSG
2502861

Query 653
EAALQSGDLVQVLPLSTILSRSPPQSAPLAAVLEDWeeeqahaaaeaaalaasaNNYLEV 712

EAALQSGDLVQVLPLSTILSRSPPQSAPLAAVLEDWEEEQAHAAAEAAALAASANNYLEV
Sbjct 2502860
EAALQSGDLVQVLPLSTILSRSPPQSAPLAAVLEDWEEEQAHAAAEAAALAASANNYLEV
2502681

Query 713 YSRDGMTTWSMGPNAGAAVPAGM 735
YSRDGMTTWSMGPNAGAAVPAGM
Sbjct 2502680 YSRDGMTTWSMGPNAGAAVPAGM 2502612

Score = 291 bits (744), Expect = 4e-83, Method: Compositional matrix adjust.
Identities = 186/210 (89%), Positives = 186/210 (89%), Gaps = 24/210 (11%)
Frame = -1

Query 1

MAATYSLATSPLTRSTPIPFSTKRVPSGRSSGALPVPGSQARLSGAGAVALDGLEGGRDD 60

MAATYSLATSPLTRSTPIPFSTKRVPSGRSSGALPVPGSQARLSGAGAVALDGLEGGRDD
Sbjct 2505639
MAATYSLATSPLTRSTPIPFSTKRVPSGRSSGALPVPGSQARLSGAGAVALDGLEGGRDD
2505460

Query 61
WHQRYSLSTSGRRRSVASGSPSNTGSSEAKNVVMASNNVTVgfsvgasssafsdassfsf 120

WHQRYSLSTSGRRRSVASGSPSNTGSSEAKNVVMASNNVTVGFSVGASSSAFSDASSFSF
Sbjct 2505459
WHQRYSLSTSGRRRSVASGSPSNTGSSEAKNVVMASNNVTVGFSVGASSSAFSDASSFSF
2505280

Query 121   parsapagpVARPPRTGVSLLSRALSTGEK------------------------EQDLLA
156
PARSAPAGPVARPPRTGVSLLSRALSTGEK   EQDLLA
Sbjct 2505279
PARSAPAGPVARPPRTGVSLLSRALSTGEKVGPEARSVFQRQLGSSLPLFYLSQEQDLLA
2505100

Query 157   HLPPVLPSTDAADPLLFFPGIPLELLREAQ 186
HLPPVLPSTDAADPLLFFPGIPLELLREAQ
Sbjct 2505099 HLPPVLPSTDAADPLLFFPGIPLELLREAQ 2505010

Score = 85.1 bits (209), Expect = 4e-16, Method: Compositional matrix adjust.
Identities = 38/50 (76%), Positives = 42/50 (84%), Gaps = 3/50 (6%)
Frame = -3

Query 499
VEVQIRTHKMHYIAEYGFAAHWKYKEQMDSEDEWLEKETQYKRWLTQYKL 548
+VQIRTHKMHYIAEYGFAAHWKYKEQMDSEDEWLEK   +R L+ Y +
Sbjct 2503453
AQVQIRTHKMHYIAEYGFAAHWKYKEQMDSEDEWLEK---VRRRLSYYAV 2503313

>jgi|chromosome_14 Creinhardtii|281|v5.5
Length=4157777

Score = 50.4 bits (119), Expect = 2e-05, Method: Compositional matrix adjust.
Identities = 22/34 (65%), Positives = 26/34 (76%), Gaps = 0/34 (0%)
Frame = +2

```
Query  491      VYGEGDVPVEVQIRTHKMHYIAEYGFAAHWKYKE  524
                V G G +P+EVQI+T  MH +AEYG AAHW YKE
Sbjct  1980728  VRGPGGIPMEVQIKTSSMHELAEYGAAAHWVYKE  1980829
```

Lambda K H
0.319 0.133 0.398

Gapped
Lambda K H
0.267 0.0410 0.140

Effective search space used: 28325807235

Database: Creinhardtii_281.genome
Posted date: Nov 13, 2018 4:44 PM
Number of letters in database: 111,100,715
Number of sequences in database: 54

Matrix: BLOSUM62
Gap Penalties: Existence: 11, Extension: 1
Neighboring words threshold: 13
Window for multiple hits: 40
