# Supplementary material for: Identification of a RelA/SpoT Homolog and Its Possible Role in the Accumulation of Astaxanthin in Haematococcus pluvialis
Source: Front Plant Sci. 2022 Feb 9;13:796997. doi: 10.3389/fpls.2022.796997 (PMC8863741; doi:10.3389/fpls.2022.796997)
Supplement: Supplementary file 2 [file Table_1.DOCX]

**Supplemental Table S1.** BG-11 medium composition

| **Composition** | **Concentration (g/L)** |
| --- | --- |
| NaNO_3_ | 1.5 |
| K_2_HPO_4_ | 0.004 |
| MgSO_4_·7H_2_O | 0.075 |
| CaCl_2_·2H_2_O | 0.027 |
| Citric acid | 0.006 |
| Ferric citrate | 0.006 |
| EDTANa_2_ | 0.001 |
| Na_2_CO_3_ | 0.02 |
| A5 (microelements) | 1 mL |
| A5 stock solution | |
| H_3_BO_3_ (2.86 g/L dH_2_O) | 2.86 |
| MnCl_2_·4H_2_O (1.86 g/L dH_2_O) | 1.81 |
| ZnSO_4_·7H_2_O (0.22 g/L dH_2_O) | 0.222 |
| Na_2_MoO_4_·2H_2_O (0.39 g/L dH_2_O) | 0.391 |
| CuSO_4_·5H_2_O (0.08 g/L dH_2_O) | 0.079 |
| Co(NO_3_)_2_·6H_2_O (0.05 g/L dH_2_O) | 0.050 |
|  | To 1.0 liter |

**Supplemental Table S2.** Primers used in this study.

| **Manipulation** | **Primer** | **Sequence (5’ to 3’)** |
| --- | --- | --- |
| Gene isolation | eHpRSH F | AAC***GGATCC***ATGAATCTGGGGCCCGTGTCC |
|  | eHpRSH R | TAA***CTCGAG***TCATGATGAAGCTGCCTGTG |
|  | 5’RACE-R1 | AGAGCGACCAAGGCCGACAGGCTTGC |
|  | 5’RACE-R2 | GCTGGACACGGGCCCCAGATTCAT |
| qRT-PCR | qHpRSH F | TCGGAAATTAGCTCGCTTTATCG |
|  | qHpRSH R | CGCCTCACACTCCTCTGGGT |
|  | qHpGGPPS F | TTGCAGCTCCTATTACCCACCC |
|  | qHpGGPPS R | CAAACAACCTGTCAACCCCTCTATC |
|  | qHpGPPS F | AAGGGTGTGGAGGGGAAACG |
|  | qHpGPPS R | GGAGCAAGCTGGCAACATGAAT |
|  | qHpFPPS F | AAGAAAAGGTTGGGCTGATTGCG |
|  | qHpFPPS R | CGGGAAGGTAGAAGGAGTAGTAGGC |
|  | qHpLCYB F | TCCAACAACATCCGCATCCAC |
|  | qHpLCYB R | CCCTAGTTCTTCCCACTCCAGTCAC |
|  | qHpCHYB F | CAACATCATGTGCGATTGGAGG |
|  | qHpCHYB R | ACAAATAGAATATGGGCGGAGCC |
|  | qHpAct F | CACCACTGCTGAGCGGGAGATA |
|  | qHpAct R | GGAACATTGTAGTCCCGCCTGA |

Restriction sites are shown in bold italics.

**Supplemental Fig. S1.** 5’ RACE amplification of the *Hprsh* mRNA. Lane 1 to 2: 1^st^ and 2^nd^ RACE PCR reactions, respectively. The 5’ end of *Hprsh* mRNA is 189 bp in length.


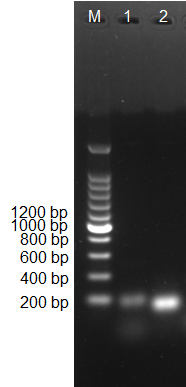


**Supplemental Fig. S2.** SDS-PAGE analysis of the recombinant HpRSH expressed in *E. coli* BL21(DE3). Arrow indicates the recombinant HpRSH protein band with a theoretical molecular weight of 85.9 kDa. P, bacterial precipitate; L, bacterial lysis; E, elution.


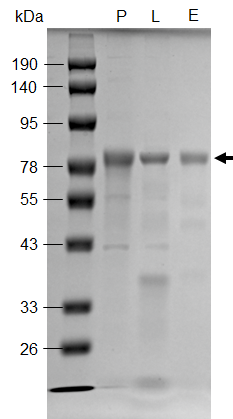


**Supplemental Fig. S3.** Morphological transformation of *H. pluvialis* after treatment with SHX, Vegetable cells indicate mobile green algae; spores are non-mobile red cysts.

**
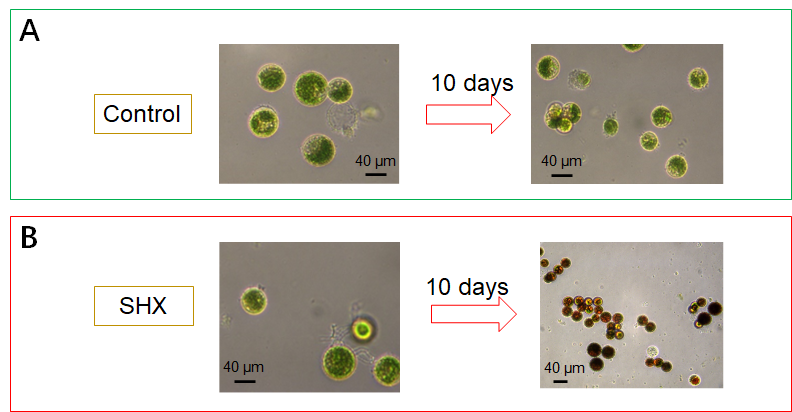
**

**Supplemental Fig. S4.** The accumulation of astaxanthin under dark condition. Error bars indicate the standard deviation (SD) of the mean (n = 3) for astaxanthin determination. Control: algal cells were cultured under dark condition for 72 h; SHX: SHX was added to the algal culture medium at a final concentration of 1 g/L, then transferred to dark condition for 72 h.

**a
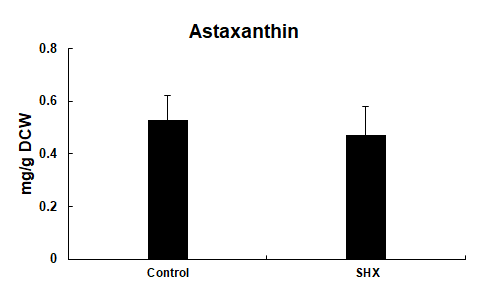
**
